# LncRNA HOXA-AS2 regulates microglial polarization via recruitment of PRC2 and epigenetic modification of PGC-1α expression

**DOI:** 10.1186/s12974-021-02267-z

**Published:** 2021-09-12

**Authors:** Xiaodong Yang, Yi Zhang, Yimeng Chen, Xiaoqin He, Yiwei Qian, Shaoqing Xu, Chao Gao, Chengjun Mo, Shengdi Chen, Qin Xiao

**Affiliations:** Department of Neurology, Ruijin Hospital, Shanghai Jiao Tong University School of Medicine, 197 Ruijin Er Road, 200025 Shanghai, PR China

**Keywords:** Parkinson’s disease, RNA-Seq, LncRNA HOXA-AS2, Microglia polarization, PRC2

## Abstract

**Background:**

Microglia-mediated neuroinflammation plays an important role in Parkinson’s disease (PD), and it exerts proinflammatory or anti-inflammatory effects depending on the M1/M2 polarization phenotype. Hence, promoting microglia toward the anti-inflammatory M2 phenotype is a potential therapeutic approach for PD. Long noncoding RNAs (lncRNAs) are crucial in the progression of neurodegenerative diseases, but little is known about their role in microglial polarization in PD.

**Methods:**

In our study, we profiled the expression of lncRNAs in the peripheral blood mononuclear cells (PBMCs) of PD patients using a microarray. RT-qPCR was used to evaluate the lncRNA levels and mRNA levels of cytokines and microglial cell markers both in vitro and in vivo. RIP and ChIP assays were analyzed for the underlying mechanism of lncRNA regulating microglial polarization.

**Results:**

We found that HOXA-AS2 was upregulated in the PBMCs of PD patients and negatively associated with peroxisome proliferator-activated receptor gamma coactivator-1a (PGC-1α) expression. Moreover, HOXA-AS2 knockdown significantly repressed microglial M1 polarization and promoted M2 polarization by regulating PGC-1α expression. Mechanistic investigations demonstrated that HOXA-AS2 could directly interact with polycomb repressive complex 2 (PRC2) and modulate the histone methylation of the promoter of PGC-1α.

**Conclusions:**

Our findings identify the upregulated lncRNA HOXA-AS2 promotes neuroinflammation by regulating microglial polarization through interacts with the PRC2 complex and epigenetically silencing PGC-1α. HOXA-AS2 may be a potential therapeutic target for microglia-mediated neuroinflammation in patients with PD.

**Supplementary Information:**

The online version contains supplementary material available at 10.1186/s12974-021-02267-z.

## Background

Parkinson’s disease (PD) is a neurodegenerative disorder caused by genetic and environmental factors that result in the progressive loss of dopaminergic (DA) neurons in the substantia nigra (SN) of the midbrain [1]. Despite intensive research, the molecular mechanisms of PD are still unknown. Microglia-mediated neuroinflammation plays an important role in the pathogenesis of PD and is inversely related to the survival of DA neurons [2]. Microglia, the innate immune cells of the central nervous system (CNS), can be activated and polarized to take on different phenotypes, including pro-inflammatory (M1) and anti-inflammatory (M2) phenotypes. M1-polarized microglia promote neuroinflammation and increase oxidative stress and neuronal injury, whereas M2-polarized microglia increase the production of anti-inflammatory factors and promote neuronal survival [3]. Based on a previous NSAID study, blocking inflammation by inhibiting M1 activation alone is unlikely to have an overall beneficial effect [4]. Therefore, promoting microglia toward the M2 phenotype is a potential therapeutic approach to suppress neuroinflammation.

The M1 and M2 activation state paradigms share phenotypic characteristics with peripheral monocytes. The developmental origin of microglia is thought to be similar to that of monocytes and macrophages, and microglia and macrophages use remarkably similar transcription factors during development [3, 5–7]. In extreme cases such as inflammation and damage, peripheral immune cells will enter the brain in large numbers [8, 9]. Studies have also demonstrated that peripheral macrophages are changed in PD and involved in the pathogenesis of PD [10–13]. Thus, studies on peripheral cells will provide interesting clues to reflect local microglial changes in the brain.

Peroxisome proliferator-activated receptor gamma coactivator-1a (PGC-1α) is a transcription coactivator for nuclear receptors and plays a key integratory role in the transcriptional control of cellular energy metabolism and mitochondrial function [14]. Studies have indicated that PGC-1α impairment plays a role in the pathogenesis of PD [15]. Recently, we found that PGC-1α could regulate microglial M1/M2 polarization, and PGC-1α expression was decreased in the peripheral blood of PD patients and was negatively correlated with disease severity [16, 17]. The expression of PGC-1α is under tight and multilayered regulation. Long noncoding RNAs (lncRNAs) are a class of RNAs greater than 200 bp in length with no protein-coding capacity that can regulate the function of target genes in the human genome through epigenetic, transcriptional, and posttranscriptional mechanisms. Increasing evidence has shown that lncRNAs are involved in multiple biological and pathological processes, including neurodegenerative disease [18, 19]. LncRNAs can regulate microglia/macrophage polarization and innate immune responses [20, 21]. A recent study demonstrated that lncRNA Tug1 could regulate PGC-1α expression in mitochondrial bioenergetics [22]. However, the role of lncRNAs in the regulation of PGC-1α in microglial polarization remains largely unknown, and more detailed functional studies are needed to unravel the biological roles of lncRNAs in PD.

In this study, we profiled the expression of lncRNAs in peripheral blood mononuclear cells (PBMCs) of PD patients using a microarray and sought to clarify whether lncRNAs are involved in microglial polarization in PD and the underlying mechanism.

## Methods

### Study participants and blood sample collection

Blood samples were collected from 5 PD patients (age: 66.20 ± 4.02; female: 2) and 4 healthy controls (age: 65.25 ± 3.77; female: 2) for microarray analysis and from 50 PD patients (age: 64.68 ± 9.82; female: 23) and 40 healthy controls (age: 64.83 ± 9.41; female: 22) for real-time quantitative PCR (RT-qPCR). There were no significant differences in age or gender between PD patients and healthy controls. All subjects were enrolled from the Movement Disorder Clinic at the Department of Neurology, Ruijin Hospital affiliated with Shanghai Jiao Tong University School of Medicine. Patients were assessed for eligibility when they were diagnosed with idiopathic Parkinson’s disease according to Parkinson’s disease Queen Square Brain Bank criteria [23]. The control group was recruited from healthy subjects exhibiting no disease symptoms who came for a routine physical examination. Peripheral venous blood (6 ml) was collected in heparin anticoagulant tubes, and peripheral blood mononuclear cells (PBMCs) were isolated using Ficoll Plus (Solarbio, Beijing, China) density gradient centrifugation. For this, blood was diluted 1:1 with phosphate-buffered saline (PBS, Gibco, USA) before layering onto Ficoll Plus. After centrifugation for 20 min at 1200 g, PBMCs were collected from the plasma-Ficoll interphase. Cells were washed twice with PBS and counted before analysis. This study was approved by the ethics committee of Ruijin Hospital. All participants signed the informed consent.

### Microarray analysis

We performed enrichment of lncRNAs from PBMCs using the miRNeasy Micro Kit and the RNeasy Mini Kit (QIAGEN, CA, USA) according to the manufacturer’s protocols. Microarray hybridization was performed based on the manufacturer’s standard protocols. Microarray slides were sequenced on an Agilent Microarray Scanner, and the raw data were normalized by the quantile algorithm GeneSpring Software 12.6.1 (Agilent Technologies). To identify differentially expressed lncRNAs between PD patients and healthy controls, filtering criteria were applied (≥ 2-fold change, *P* < 0.05). Differentially expressed lncRNAs with statistical significance between the two groups were identified through volcano plot filtering. The microarray work was performed by Kang Cheng Biotech, Shanghai, China.

### Cell culture, stimulation, and transfection

Human monocytic THP-1 cells and mouse microglial BV2 cells were cultured in RPMI-1640 medium supplemented with 10% fetal bovine serum (Gibco, USA) at 37 °C and 5% CO_2_. To induce the polarization of macrophages, cells were treated with 100 ng/ml lipopolysaccharide (LPS) for 12 h and 20 ng/ml interleukin-4 (IL-4) for 6 h. Cells were plated 24 h prior to transfection at a confluency of 70 to 80%. The plasmid vectors were transfected using Lipofectamine™ 3000 (Invitrogen, USA) reagent according to the manufacturer’s protocol. Small RNA interference (siRNA) transfection was performed using Lipofectamine RNAiMAX (Invitrogen, USA) according to the manufacturer’s instructions. Full-length complementary cDNAs of HOXA-AS2 and PGC-1α were synthesized and cloned into the expression vector pcDNA3.1 (Invitrogen, China). HOXA-AS2 siRNA (HOXA-AS2i), PGC-1α siRNA (PGC-1αi), and scrambled negative control siRNA (Scr) were synthesized by GenePharma Co. (Shanghai, China). The siRNA sequences are listed in Supplementary Table S1.

### Real-time quantitative PCR

Total RNA was isolated from tissues or cell cultures using TRIzol reagent (Invitrogen, USA). Complementary DNA (cDNA) was synthesized using PrimeScript RT reagent (Takara, Dalian, China). RT-qPCR was performed on an Applied Biosystem 7500 Real-time PCR system (Applied Biosystems, CA, USA) using SYBR-Green Master Mix (Takara, Tokyo, Japan). Glyceraldehyde 3-phosphate dehydrogenase (GAPDH) was used as an internal control, and the RT-qPCR result was calculated by the 2-^ΔΔ^CT method. The primer sequences for all genes are provided in Supplementary Table S2.

### Western blot

The cells were homogenized in RIPA buffer (Beyotime, Shanghai, China) containing protease and phosphatase inhibitors to extract proteins. The protein samples were separated by sodium dodecyl sulfonate-polyacrylamide gel electrophoresis (SDS-PAGE) and then transferred onto polyvinylidene fluoride (PVDF) membranes. Then, the membranes were incubated with the primary antibodies. Anti-PGC-1α (sc13067, Santa Cruz, 1:1000), anti-EZH2 (ab3748, Abcam, 1:1000), and anti-β-actin (A5441, Sigma-Aldrich, 1:2500) antibodies. The protein bands were analyzed using Image Lab software (Bio-Rad), and the gray values of experimental proteins were detected using ImageJ software.

### Experimental animals and protocols

Male C57BL/6 mice (20–25 g) were purchased from Shanghai Model Organisms Center, Inc. (Shanghai, China). The mice were kept under environmentally controlled conditions (ambient temperature: 20 ± 2 °C; humidity: 50–65%) on a 12 h light/dark cycle and provided ad libitum access to food and water. The mice were substantially anesthetized and placed under a stereotaxic apparatus (David Kopf Instrument, CA, USA) for intracerebroventricular (ICV) injection into the left lateral ventricle of mice (bregma: − 2 mm, lateral: 2 mm, dorsoventral: 3 mm) using a Hamilton microsyringe. HOXA-AS2 siRNA and control siRNA were carefully diluted with equal volumes of transfection reagent siPORT NeoFX (Invitrogen, CA, USA), and the mixtures were then incubated at 25 °C for 15 min according to the guidelines supplied by the manufacturer for five consecutive days. The mice were injected intraperitoneally with LPS (1 mg/kg) after the last ICV injection. Open-field tests were carried out 4 h after LPS challenge to evaluate behavioral activity, as previously described [24].

### Fluorescence in situ hybridization and immunocytofluorescence staining

Fluorescence in situ hybridization (FISH) assays were performed using a Fluorescent In Situ Hybridization Kit (RiboBio, Guangzhou, China) according to the protocol. Probe sequences targeting HOXA-AS2 were designed and generated by RiboBio, and experiments were performed according to the manufacturer’s instructions. Immunocytofluorescence staining (ICF) was performed according to the previous report. Briefly, tissue sections were fixed, permeabilized, and incubated with primary rabbit anti-Iba-1 antibody (ab5076, Abcam, 1:800) overnight at 4 °C, followed by 2 h of incubation with FITC-conjugated secondary antibody (Jackson ImmunoResearch). Fluorescence images were captured using an Olympus laser scanning microscope FV3000 (Olympus Corporation, Japan) and analyzed using Image-Pro Plus (Media Cybernetics).

### Cytoplasmic and nuclear RNA isolation

Cytoplasmic and nuclear RNA was extracted using nuclear/cytoplasmic isolation reagent (Thermo Fisher Scientific) according to the manufacturer’s instructions. Cytoplasmic and nuclear fractions were split for RNA extraction. The expression levels of HOXA-AS2, NEAT1, and GAPDH in the nucleus or cytoplasm were detected by RT-qPCR.

### RNA immunoprecipitation and RNA pull-down assay

RNA immunoprecipitation (RIP) was performed using a Magna RIP RNA-Binding Protein Immunoprecipitation Kit (Millipore, USA) according to the manufacturer’s protocols. Cells were collected and lysed in RIP lysis buffer and then incubated with magnetic beds and anti-EZH2 (ab3748, Abcam), anti-SUZ12 (ab236322, Abcam), and anti-RbAp48 (ab1765, Abcam) antibodies overnight at 4 °C. After the precipitate was washed with RIP wash buffer six times, the coprecipitated RNA was detected by qPCR analysis. Immunoglobulin G RIP of cells served as the negative control. RNA pull-down assays were performed using the Magnetic RNA-Protein Pull-Down Kit (Thermo Scientific). The retrieved proteins were detected by Western blotting with an anti-EZH2 antibody.

### Chromatin immunoprecipitation assay

Chromatin immunoprecipitation (ChIP) assays were performed using the ChIP Assay Kit (Beyotime, China) following the manufacturer’s protocol. Briefly, cells were cross-linked with 1% formaldehyde solution for 10 min at 37 °C and quenched with 125 mM glycine. DNA fragments ranging from 200 to 500 bp were yielded via sonication. Then, the lysates were immunoprecipitated with anti-EZH2 and anti-H3K27me3 antibodies (Millipore, USA). IgG was used as negative control. The precipitate DNA was extracted and analyzed by RT-qPCR. ChIP primers are listed in Supplementary Table S2.

### Statistical analysis

Statistical analyses were performed using SPSS software v.13.0 (Chicago, IL, USA) and GraphPad Prism software (La Jolla, CA, USA). The differences between two or multiple groups were analyzed by *t* test or one-way analysis of variance (ANOVA) if the data were normally distributed. Nonparametric Mann-Whitney *U* or Kruskal-Wallis tests were used for continuous variables if the data were not normally distributed. Spearman’s correlation coefficient was used for correlation analysis. Receiver operating characteristic (ROC) curves were constructed to detect biomarkers for differentiating PD from healthy controls; the predictive accuracies of the parameters for detecting PD were quantified by using the area under the curve (AUC). Data are shown as the mean ± SD. Statistical significance was defined as *P* < 0.05.

## Results

### HOXA-AS2 is involved in microglial polarization in PD

**Fig. 1:**
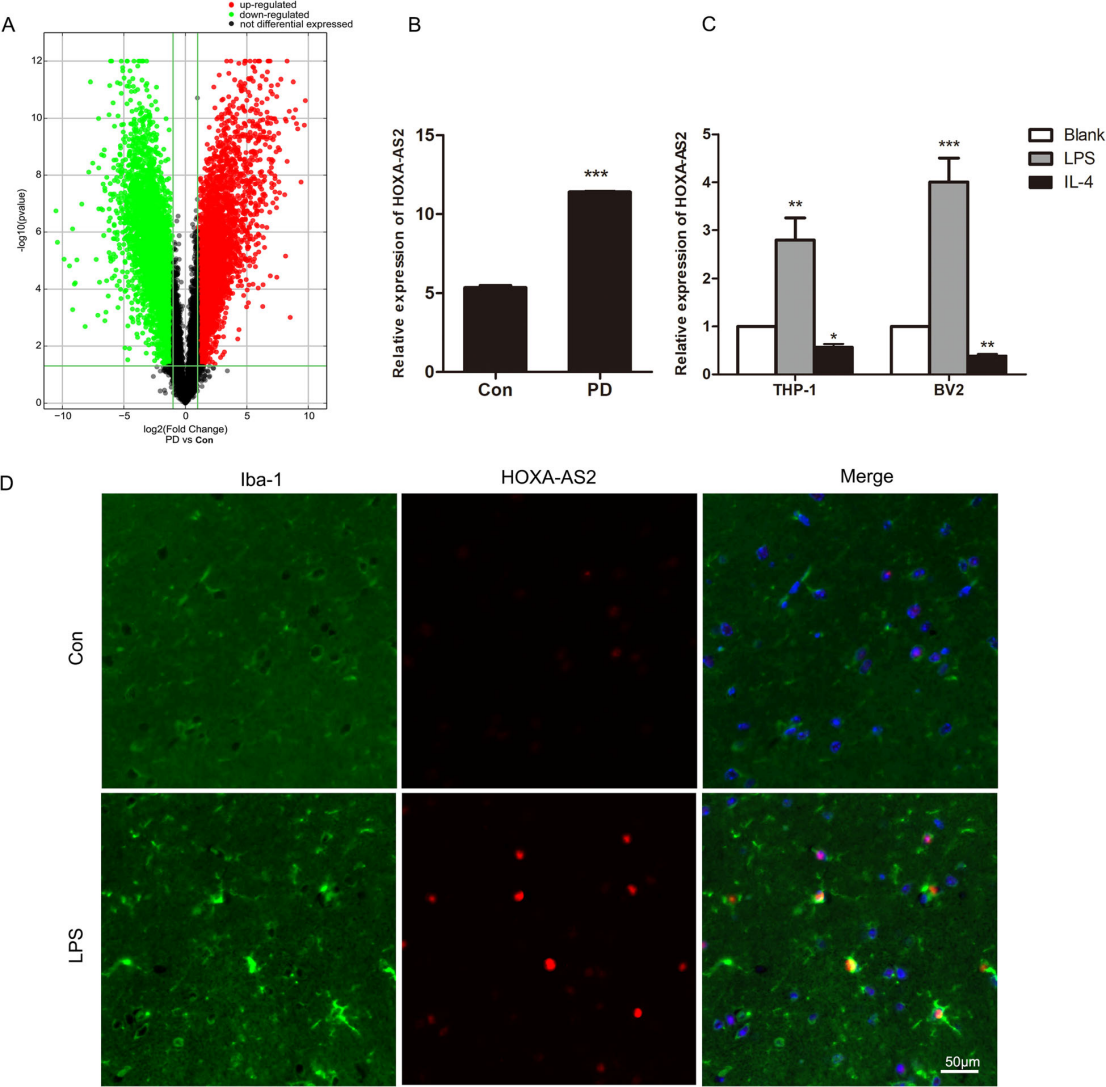
HOXA-AS2 is upregulated in the PBMCs of PD patients and in M1-polarized microglia. **A** The volcano figure based on lncRNAs showing differential expression patterns in PBMCs of PD patients and healthy controls. **B** HOXA-AS2 expression in PBMCs of PD patients and healthy controls. **C** RT-qPCR analysis of HOXA-AS2 in THP-1 or BV2 microglia treated with LPS or IL-4 versus the untreated control. **D** Representative FISH analysis of HOXA-AS2 (red) costained with anti-Iba1 antibody (green) in substantia nigra sections from LPS-treated mice. ^*^P < 0.05, ^**^P < 0.01, ^***^P < 0.001 versus control group (one-way ANOVA)

To identify potential lncRNAs participating in the regulation of microglial polarization in PD patients, we performed RNA-sequencing (RNA-seq) analysis to detect differentially expressed lncRNAs in PBMCs from 5 PD patients and 4 healthy controls. In the discovery phase, our analysis of transcripts classified as noncoding RNAs revealed several differentially regulated noncoding RNAs (Fig. 1A). Among them, HOXA cluster antisense RNA 2 (HOXA-AS2) was one of the most abundantly expressed lncRNAs, which was significantly increased in PD patients (5.35 ± 0.27 vs. 11.39 ± 0.07, Fig. 1B) and has been found to be associated with multiple inflammation-linked cancers [25]. This information prompted us to examine whether HOXA-AS2 is involved in the regulation of microglial polarization in PD. First, we validated microarray results and examined HOXA-AS2 levels during microglial polarization using RT-qPCR in human THP-1 cells and mouse BV2 microglial cells following LPS or IL-4 stimulation. We found that HOXA-AS2 expression was increased after LPS stimulation and decreased in IL-4-stimulated cells (Fig. 1C). Consistent with our in vitro experimental results, a marked increase in the HOXA-AS2 signal was detected in Iba1-positive microglia, which tended to be M1 polarized from LPS-treated mice (Fig. 1D). These data revealed that the HOXA-AS2 level can vary dynamically according to the microglial polarization state and that the function of HOXA-AS2 in humans and mice is conserved.

### HOXA-AS2 promotes microglial M1 polarization and suppresses M2 polarization

**Fig. 2:**
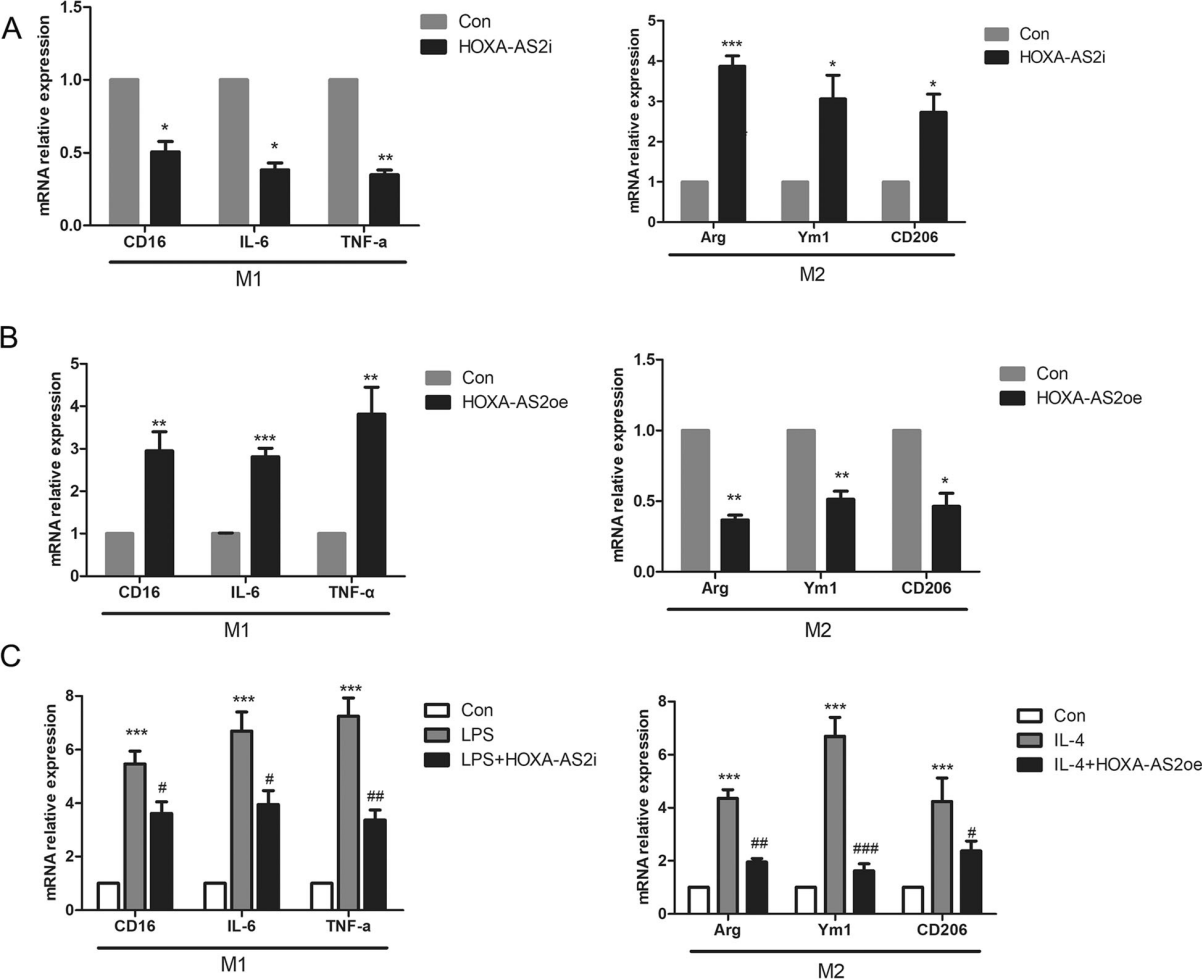
HOXA-AS2 promotes microglial M1 polarization and suppresses M2 polarization. **A** RT-qPCR analysis of M1 and M2 markers in microglia transfected with HOXA-AS2 small RNA (HOXA-AS2i) versus the control. **B** RT-qPCR analysis of M1 and M2 markers in microglia transfected with the HOXA-AS2 overexpression plasmid (HOXA-AS2oe) versus the control. **C** RT-qPCR analysis of M1 and M2 markers in LPS- or IL-4-stimulated BV2 cells transfected with HOXA-AS2oe or HOXA-AS2i. ^*^P < 0.05, ^**^P < 0.01, ^***^P < 0.001 versus control group. ^#^*P* < 0.05, ^##^*P* < 0.01, ^###^*P* < 0.001 versus LPS- or IL-4-stimulated only groups (one-way ANOVA). The data are from three independent experiments and represented as mean ± SD

**Fig. 3:**
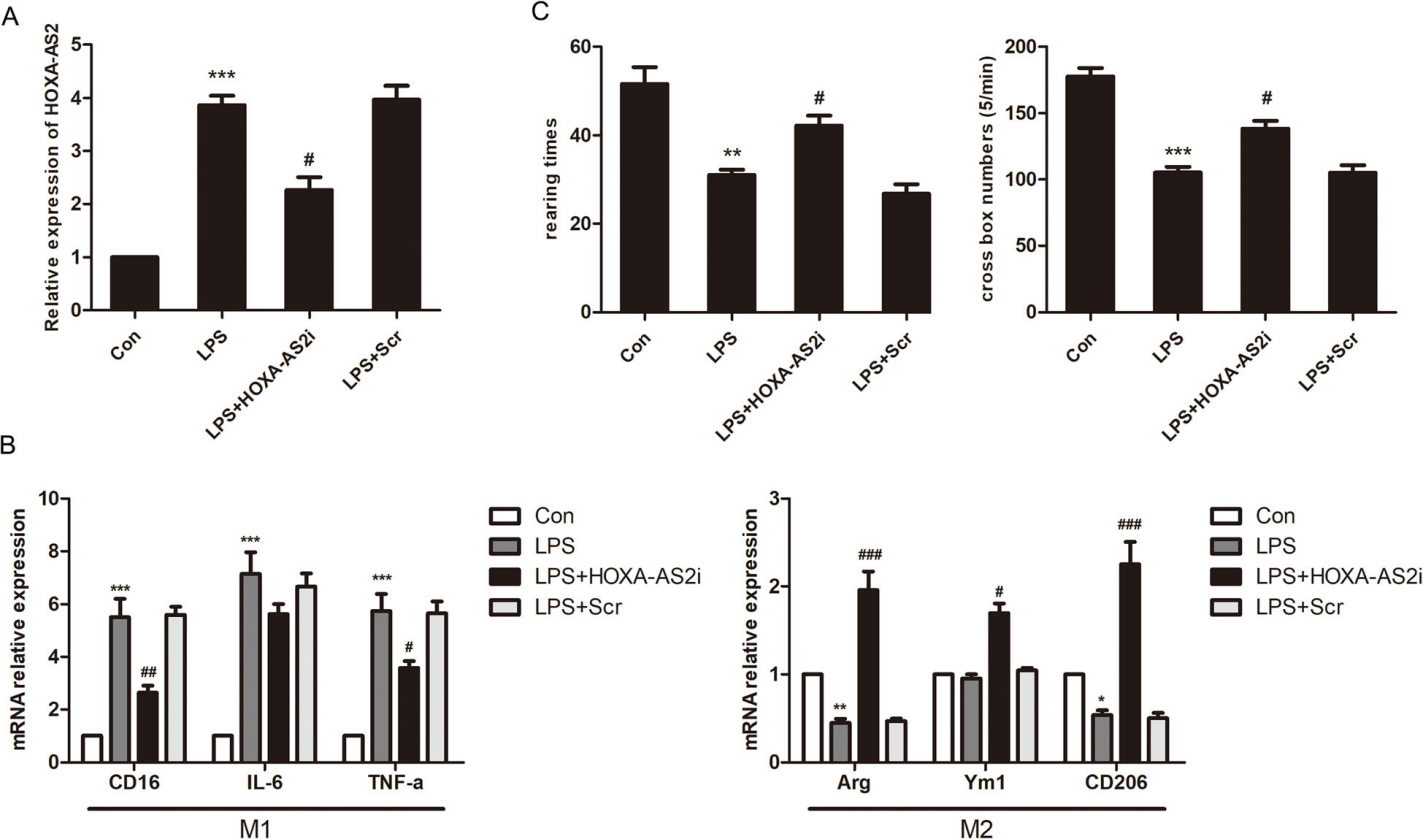
Interfering with HOXA-AS2 suppresses LPS-induced M1 microglial polarization, promotes polarization to the M2 phenotype, and ameliorates impaired behaviors in LPS-injected mice. **A** Effects of intraventricular injection of HOXA-AS2 siRNA on HOXA-AS2 mRNA expression. **B** Effects of intraventricular injection of HOXA-AS2 siRNA on M1 and M2 markers in the substantia nigra as determined by RT-qPCR in LPS-injected mice. **C** Effects of intraventricular injection of HOXA-AS2 siRNA on LPS-induced impaired behavior as determined by open-field tests where the number of times the mice stood on their hind legs (rearings) and the number of squares crossed by the mice were recorded. ^*^P < 0.05, ^**^P < 0.01, ^***^P < 0.001 versus control group. ^#^*P* < 0.05, ^##^*P* < 0.01, ^###^*P* < 0.001 versus the LPS-injected only group (*n* = 8/group, one-way ANOVA). Data are represented as the mean ± SD

To investigate whether differential expression of HOXA-AS2 directly regulates microglial polarization, we transfected BV2 cells with HOXA-AS2 overexpression plasmid (HOXA-AS2oe) or a small interfering RNA targeting HOXA-AS2 (HOXA-AS2i). We found that the HOXA-AS2 levels were significantly upregulated by the HOXA-AS2 mimic and significantly downregulated by the HOXA-AS2 inhibitor compared with the corresponding controls (Supplement Fig. 1). RT-qPCR results showed that interference with HOXA-AS2 in BV2 cells resulted in a decrease in the expression of M1 markers CD16, interleukin-6 (IL-6), and tumor necrosis factor-α (TNF-α) and significantly elevated levels of M2-associated gene expression of arginase (Arg), Ym1, and CD206 (Fig. 2A). Conversely, HOXA-AS2 overexpression in BV2 cells increased the expression of M1 markers and decreased the expression of M2 markers (Fig. 2B). Additionally, lncRNA HOXA-AS2 interference suppressed LPS-induced M1 polarization, while HOXA-AS2 overexpression suppressed IL-4-induced M2 polarization (Fig. 2C). We further investigated the specific role of HOXA-AS2 in microglial polarization in vivo. We injected HOXA-AS2 siRNA or scramble siRNA into the ventricles of mice followed by stimulation with LPS. The expression level of HOXA-AS2 in the SN of mice was significantly decreased after HOXA-AS2 siRNA treatment (Fig. 3A). LPS injection increased the expression of microglial M1 markers and reduced the expression of anti-inflammatory M2 markers (Arg and CD206), while HOXA-AS2 knockdown halted M1 responses (CD16 and TNF-α) and promoted microglial polarization to the protective M2 phenotype (Fig. 3B). HOXA-AS2 knockdown also improved ambulation and rearing behavior of the mice compared with LPS-treated only mice, as determined by the open-field test (Fig. 3C). These results indicate that HOXA-AS2 plays an important role in microglial polarization and that lncRNA HOXA-AS2 promotes microglial M1 polarization and suppresses M2 polarization.

### HOXA-AS2 suppresses PGC-1α transcription to regulate microglial polarization

**Fig. 4:**
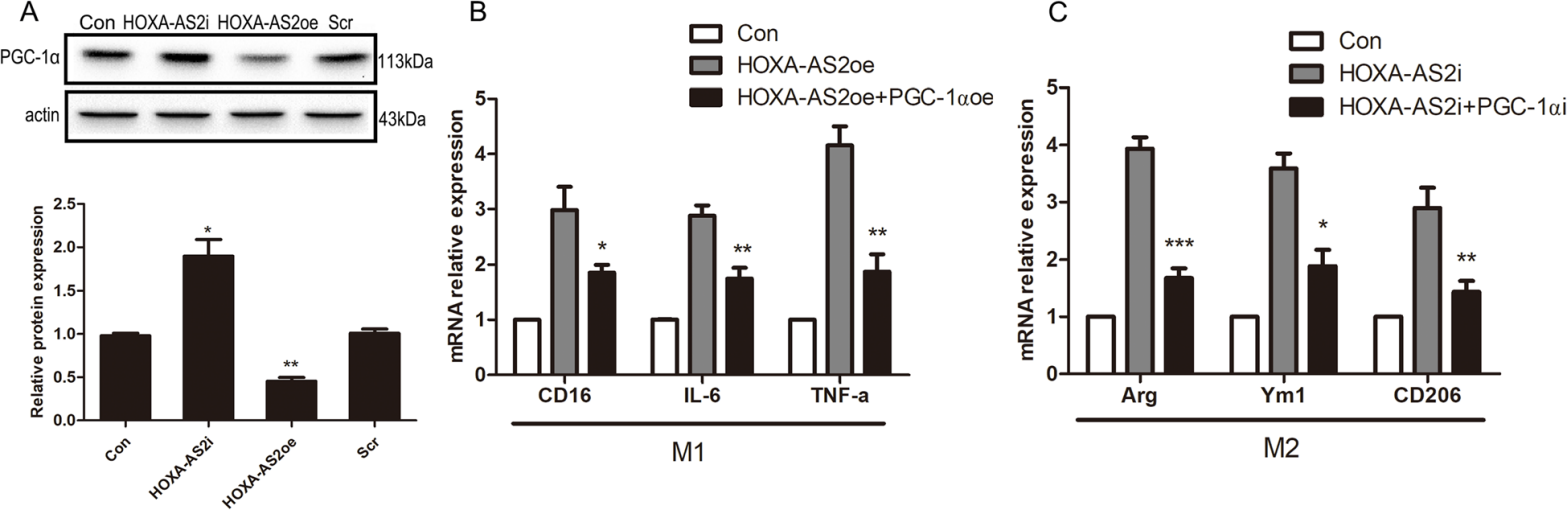
HOXA-AS2 suppresses PGC-1α transcription to regulate microglial polarization. **A** Representative Western blotting showing PGC-1α protein levels in BV2 cells transfected with HOXA-AS2 plasmid or HOXA-AS2i. (^*^P < 0.05, ^**^P < 0.01 versus control group, Student’s *t* test). **B** RT-qPCR analysis of M1 markers in BV2 cells transfected with HOXA-AS2 and PGC-1α plasmids. **C** RT-qPCR analysis of M2 markers in BV2 cells transfected with HOXA-AS2i and PGC-1αi. ^*^P < 0.05, ^**^P < 0.01, ^***^P < 0.001 versus HOXA-AS2oe or HOXA-AS2i groups (one-way ANOVA). The data are from three independent experiments and represented as mean ± SD

We next attempted to elucidate the mechanism by which HOXA-AS2 exerts its modulatory effect on microglial polarization. LncRNAs are known to influence the biology of different cell types by regulating the expression of multiple genes. PGC-1α is an important factor that can regulate microglial M1/M2 polarization, and its expression is significantly reduced in PD patients. We next focused our efforts on establishing a potential link between HOXA-AS2 and PGC-1α. We first tested whether PGC-1α levels were affected by HOXA-AS2 and found that HOXA-AS2i could increase PGC-1α expression, while PGC-1α was significantly reduced in HOXA-AS2oe BV2 cells (Fig. 4A). To test whether PGC-1α is necessary to mediate the effect of HOXA-AS2 on microglial polarization, we performed gain/loss-of-function experiments targeting PGC-1α levels in HOXA-AS2-oe/i cells. We found that PGC-1αoe partially prevented HOXA-AS2oe-mediated M1 microglial polarization (Fig. 4B). Conversely, knockdown of PGC-1α abolished HOXA-AS2i-mediated M2 microglial polarization (Fig. 4C). Taken together, these data suggest that the HOXA-AS2/PGC-1α axis is important in the regulation of microglial polarization.

### HOXA-AS2 binds to PRC2 and epigenetically suppresses PGC-1α gene expression

**Fig. 5:**
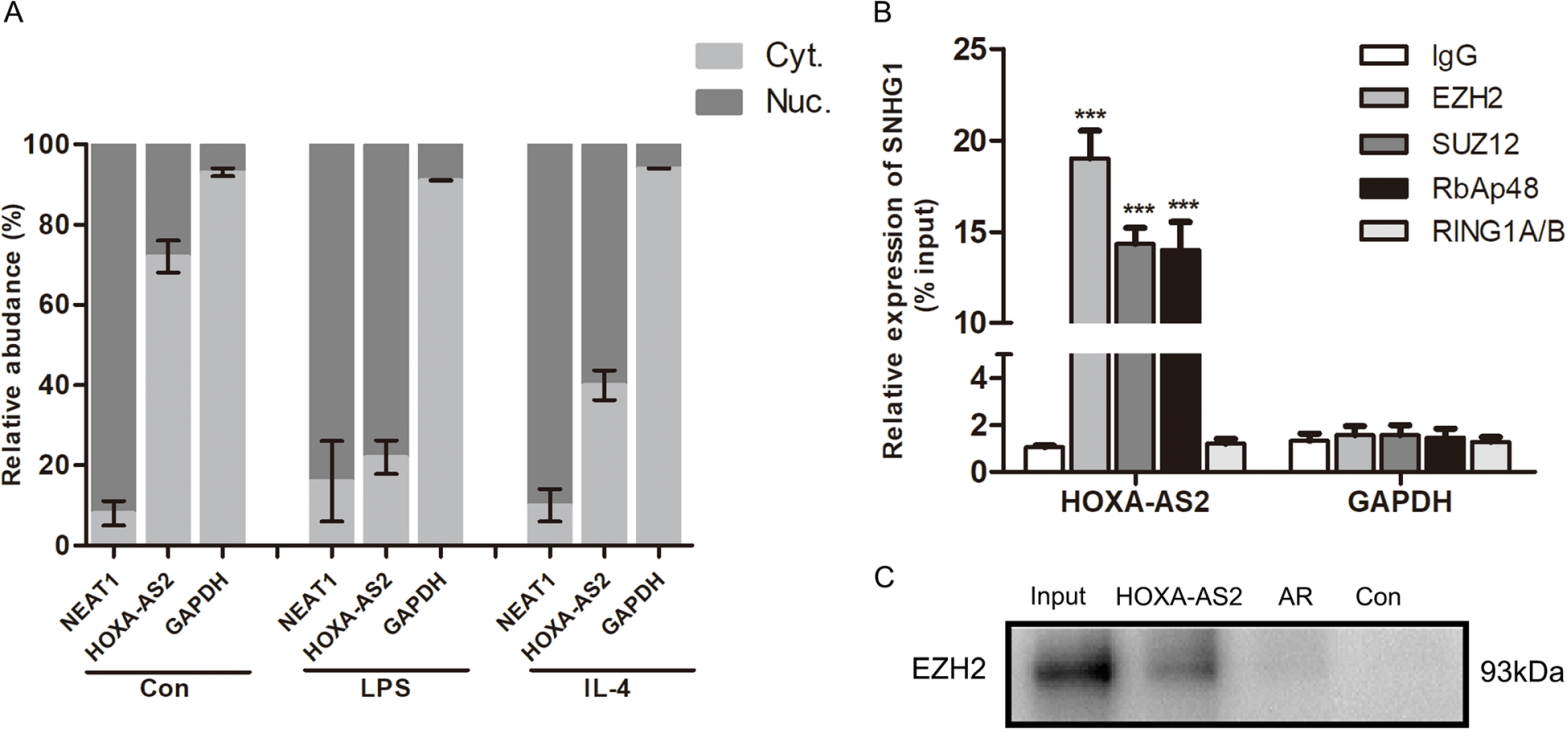
HOXA-AS2 acts as a modular scaffold of PRC2 in the nucleus. **A** Relative HOXA-AS2 expression levels in nuclear and cytosolic fractions. Nuclear controls: NEAT1; cytosolic controls: GAPDH. **B** RIP experiments for EZH2, SUZ12, RbAp48, and RING1A/B were performed, and the coprecipitated RNAs were subjected to RT-qPCR for HOXA-AS2. GAPDH was employed as a negative control. **C** RNA pulldown was used to examine the direct interaction between HOXA-AS2 and EZH2 in BV2 cells. The EZH2 protein levels were evaluated by Western blot. AR (Antisense RNA) was used as a positive control. ^***^P < 0.001 versus control group (Student’s *t* test). The data are from three independent experiments and represented as mean ± SD

**Fig. 6:**
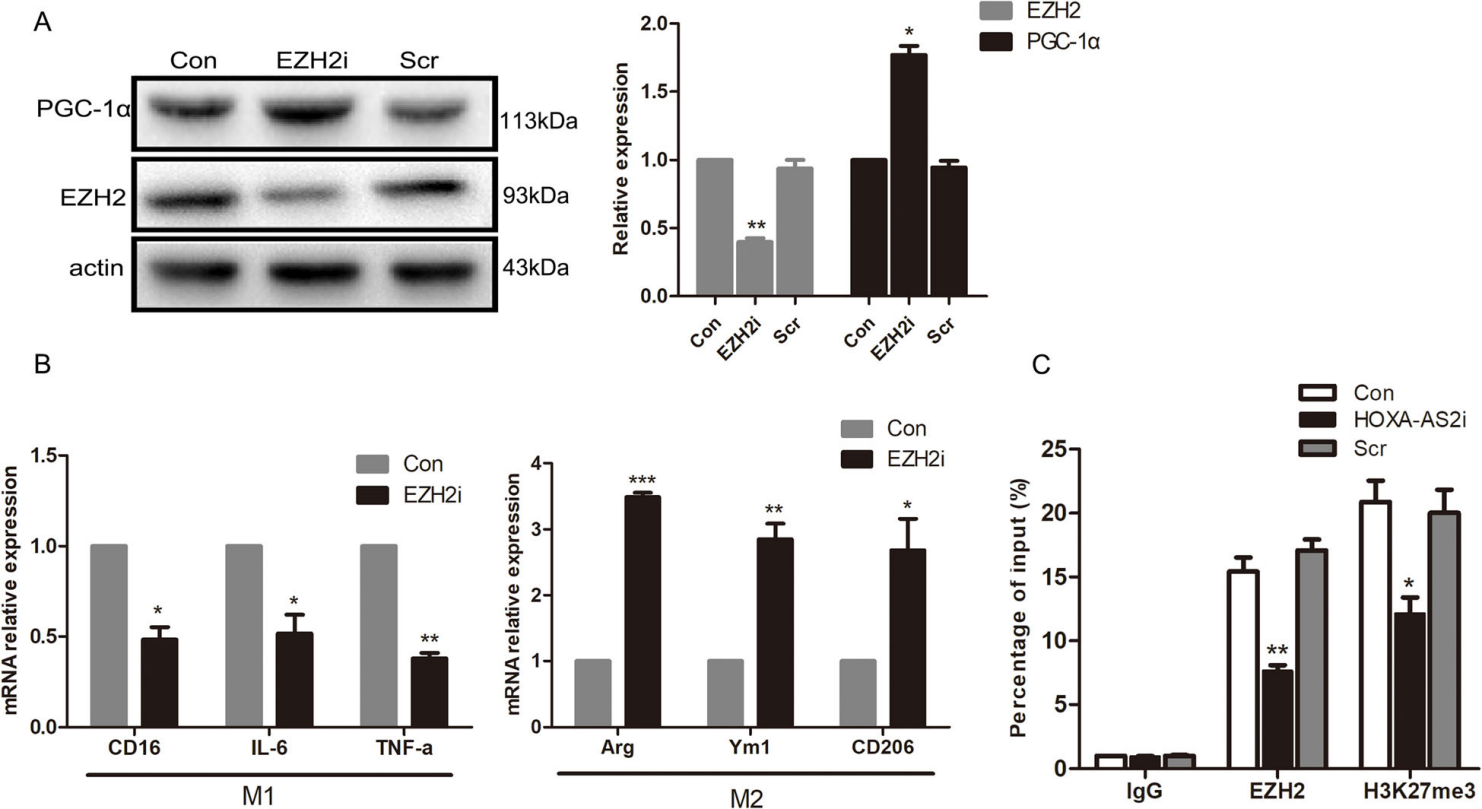
HOXA-AS2 participates in the epigenetic repression of PGC-1α by interacting with PRC2. **A** Representative Western blotting showing PGC-1α and EZH2 protein levels in BV2 cells transfected with EZH2i. **B** RT-qPCR analysis of M1 and M2 markers in BV2 cells transfected with EZH2i. **C** ChIP assays were performed to detect EZH2 and H3K27me3 occupancy in the PGC-1α promoter region. Enrichment of DNA was quantified relative to the amount of input by RT-qPCR. IgG was used as a negative control. ^*^P < 0.05, ^**^P < 0.01, ^***^P < 0.001 versus control group (Student’s *t* test). The data are from three independent experiments and represented as mean ± SD

We wanted to determine how HOXA-AS2 regulates PGC-1α expression. Recent studies have shown that a significant number of lncRNAs exert their activity in cooperation with chromatin-modifying enzymes to promote epigenetic activation or silencing of gene expression. Specifically, lncRNAs can recruit chromatin remodeling complexes, including polycomb repressive complex 1 (PRC1) and PRC2, to the promoter region of target genes repressing transcription to modulate downstream gene expression, serving as scaffolds for the histone modification complex [26]. We used a bioinformatics algorithm with the RNA-protein interaction prediction software RPISeq (http://pridb.gdcb.iastate.edu/RPISeq/references.php). HOXA-AS2 was predicted to interact with a panel of chromatin modifiers, including EZH2, SUZ12, and RbAp48, which are the main catalytic subunits of PRC2 (as the RF or SVM score > 0.5, supplement Fig. 2). To verify our hypothesis, we first examined HOXA-AS2 subcellular localization given that the function of one lncRNA generally depended on its subcellular distribution. We found that LPS or IL-4 treatment sharply increased the percentage of nuclear distribution of HOXA-AS2 in BV2 cells (Fig. 5A). We then performed an RNA immunoprecipitation assay with antibodies against EZH2, SUZ12, and RbAp48. As shown in Fig. 5B, a high proportion of HOXA-AS2 was observed to bind with EZH2, SUZ12, and RbAp48 relative to the immunoglobulin G (IgG) control. The RNA pull-down experiment confirmed the reverse binding between HOXA-AS2 and EZH2 (Fig. 5C). However, no detectable interaction between RING1A/B (subunits of PRC1) and HOXA-AS2 was found. PGC-1α expression was increased after EZH2 knockdown (Fig. 6A), concomitant with significantly upregulated expression of M2 markers and decreased expression of M1 markers (Fig. 6B). Moreover, we performed a ChIP assay to investigate whether the interaction between EZH2 and the promoter areas of PGC-1α was affected by HOXA-AS2. The results showed that HOXA-AS2 silencing significantly decreased the binding activity between EZH2 and the PGC-1α promoter. Furthermore, the levels of H3K27me3 at the PGC-1α promoter were changed accordingly (Fig. 6C). Collectively, these studies strongly suggest that HOXA-AS2 recruits PRC2 to the PGC-1α promoter and suppresses PGC-1α transcription by altering the epigenetic landscape, thereby inhibiting microglial M2 polarization.

### Increased levels of HOXA-AS2 in PBMCs from patients with PD

**Fig. 7:**
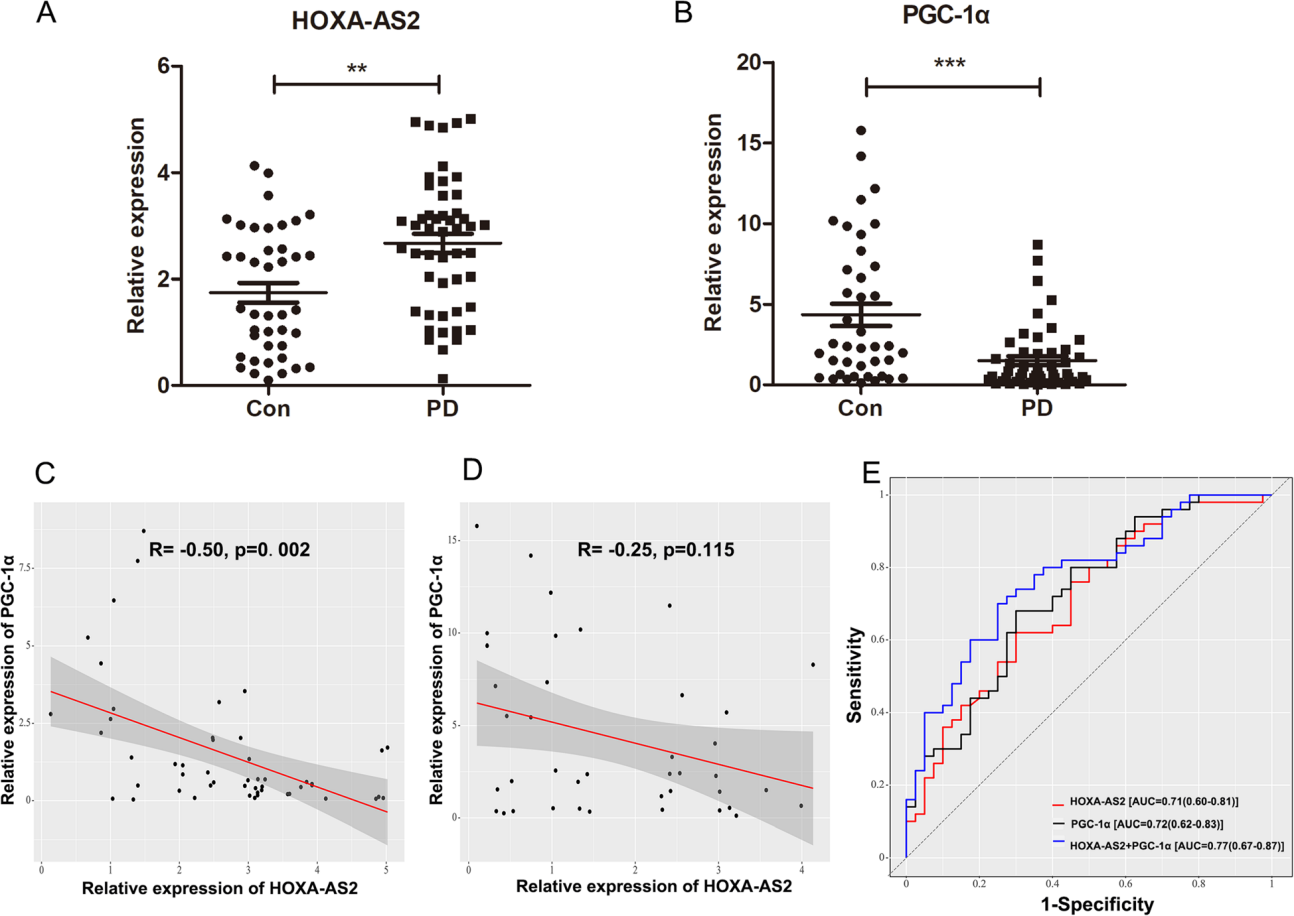
The HOXA-AS2 levels are increased in the PBMCs of patients with PD and show strong diagnostic value. **A**, **B** RT-qPCR analysis of the expression levels of HOXA-AS2 and PGC-1α in PBMCs from 50 PD patients and 40 healthy controls. ^**^P < 0.01, ^***^P < 0.001 (one-way ANOVA, data are represented as the mean ± SD). **C**, **D** Correlation analysis of the expression levels of HOXA-AS2 and PGC-1α in PD patients and healthy controls. **E** Receiver operating curve (ROC) analysis of HOXA-AS2 and PGC-1α levels in the diagnosis of PD. AUC indicates area under the ROC curve

To determine the potential importance of HOXA-AS2 in Parkinson’s disease, we evaluated the expression of HOXA-AS2 and PGC-1α in PBMCs from 50 PD patients and 40 healthy controls with RT-qPCR. A similar result was observed: HOXA-AS2 was significantly increased in patients with PD compared with healthy subjects (1.73 ± 1.19 vs. 2.66 ± 1.24, Fig. 7A). Similar to our previous study, PGC-1α levels were decreased in PD patients (4.35 ± 4.38 vs. 1.51 ± 1.98, Fig. 7B). Moreover, correlation analysis revealed that HOXA-AS2 expression was negatively associated with PGC-1α expression in PD patients (*R* = − 0.50, *P* = 0.002, Fig. 7C), which did not occur in healthy controls (*R* = − 0.25, *P* = 0.115, Fig. 7D). Moreover, in the ROC analysis, the combination of HOXA-AS2 and PGC-1α showed a higher predictive value (AUC = 0.77, specificity = 75%, sensitivity = 70%) for PD and healthy controls than any individual marker (HOXA-AS2: AUC = 0.71, specificity = 70%, sensitivity = 62%; PGC-1α: AUC = 0.72, specificity = 70%, sensitivity = 68%) (Fig. 7E).

## Discussion

**Fig. 8:**
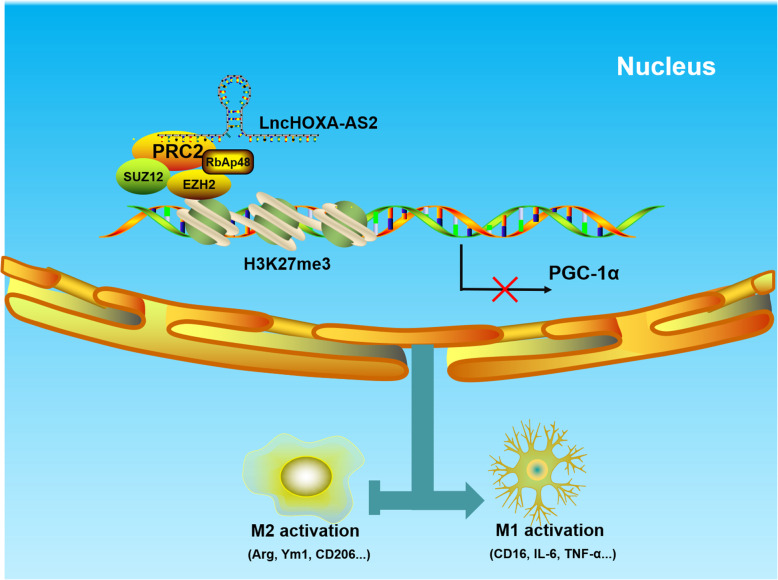
Schematic model of the proposed mechanism by which HOXA-AS2 regulates microglial polarization

The key finding of this study is that HOXA-AS2 plays an important role in microglial polarization. The results of our study demonstrated that HOXA-AS2 was significantly upregulated in the PBMCs of PD patients and negatively associated with PGC-1α expression. Functional studies revealed that HOXA-AS2 is involved in microglial polarization by interacting with the PRC2 complex and epigenetically silencing PGC-1α (Fig. 8).

Microglia-mediated neuroinflammation is a prominent feature shared by various neurodegenerative diseases, such as Parkinson’s disease (PD), Alzheimer’s disease (AD), and amyotrophic lateral sclerosis (ALS) [27, 28]. Microglial activation is the principal component of neuroinflammation in the central nervous system (CNS), which provides the first line of defense whenever injury or disease occurs. First, proposed for macrophages, the concept of alternative immune polarization into M1 and M2 phenotypes was then transferred to activated microglia. It has been shown that neuroinflammation and dopamine neuron death induced by circulating immune cells play a crucial role in the pathogenesis of PD [10, 11]. Thus, we profiled the expression of lncRNAs in the PBMCs of PD patients and healthy controls. The preliminary experimental results showed that HOXA-AS2 was significantly upregulated in PBMCs of PD patients. Furthermore, HOXA-AS2 expression is decreased in M2-polarized microglia and increased in M1-polarized microglia. These data suggested that strategies inhibiting the expression of HOXA-AS2 may be effective in regulating microglial polarization.

HOXA-AS2 is a 1048-bp lncRNA located between the HOXA3 and HOXA4 genes in the HOXA cluster that is tightly associated with inflammation-linked cancers. Studies have shown that high expression of HOXA-AS2 may be associated with various biological processes in malignant tumors, such as apoptosis, invasion, migration, and proliferation [25]. Studies have found that HOXA-AS2 is able to inhibit endothelial inflammation via the nuclear factor kappa B (NF-κB) pathway and that lower expression of HOXA-AS2 in diabetic nephropathy is associated with kidney injury and inflammatory response [29, 30]. However, whether HOXA-AS2 plays a role in regulating microglial polarization and the molecular mechanisms involved have not been fully characterized. Here, we revealed a novel biological effect of HOXA-AS2 in microglial polarization. HOXA-AS2 promotes microglial M1 polarization and suppresses M2 polarization, while HOXA-AS2 knockdown halted M1 microglial inflammatory responses and promoted microglial polarization to the M2 protective phenotype, which was associated with improved motor performance in mice.

We further explored the underlying molecular mechanisms by which HOXA-AS2 regulated downstream effectors in microglial polarization, showing that HOXA-AS2 expression was negatively related to PGC-1α expression and that HOXA-AS2 could regulate PGC-1α expression. PGC-1α is a transcriptional coactivator of nuclear receptors and has emerged as a key molecule in M2 microglial polarization, thus playing a significant role in the onset and progression of PD [16, 31]. Reduced expression of PGC-1α in the brains of patients with PD was reported [32]. The restoration of the level of PGC-1α may represent an appealing opportunity for therapeutic treatments. A better understanding of the intricate network subtended to PGC-1α may provide helpful insights into the development of more precise and effective therapies for PD. LncRNAs can regulate the function of target genes through epigenetics, transcriptional regulation, and posttranscriptional regulation in the human genome. Among these roles, epigenetic changes caused by methylation are an important pathway to change the expression of target genes [33]. The biological function of lncRNAs is largely dependent on their subcellular localization. LncRNAs located in the nucleus can affect chromatin structure and gene transcription by recruiting related modification enzymes or transcription factors. Cytosolic lncRNAs can regulate mRNA stability and protein localization by acting as microRNA sponges. LncRNAs that localize both in the cytoplasm and the nucleus could act as multifunctional lncRNAs [34]. Recent research emphasizes that epigenomic factors play a crucial role in the development of PD, and it is assumed that the initiation of PD is promoted by a combination of a genetic predisposition and environmental triggers [35, 36]. Although several studies have demonstrated that HOXA-AS2 promotes tumor proliferation by serving as an “miRNA sponge” [30, 37, 38], it has also been shown to enhance gastric cancer proliferation by epigenetically silencing the expression of p21, plk3, and DDIT3 [39, 40]. A study highlighted the role of aberrant DNA methylation of the HOXA gene cluster in the pathogenesis of Alzheimer’s disease [41]. Recent studies have found that HOXA-AS2 could directly interact with enhancers of EZH2 and lysine-specific demethylase 1 (LSD1) involved in pancreatic cancer and acute myeloid leukemia [42, 43]. Here, we found that HOXA-AS2 exhibited nuclear retention under LPS/IL-4 treatment and participated in PRC2-mediated epigenetic silencing of PGC-1α. We hypothesize that HOXA-AS2 might perform different functions in the cytoplasm and nucleus, and the balance is likely maintained under a given condition. PRC2 is a histone methyltransferase involved in epigenetic silencing. EZH2, a methyltransferase and an important subunit of PRC2, is frequently reported to participate in many essential biological processes of epigenetic [44]. In this study, HOXA-AS2 interacted with PRC2 by binding with EZH2, SUZ12, and RbAp48; however, components of PRC1 showed no interaction with HOXA-AS2 in microglial polarization, suggesting that the regulatory effects of HOXA-AS2 on genes have tissue and cell specificity. Our experiments demonstrated that the gene-activated marker H3K27ac was more enriched in the promoter region of PGC-1α.

LncRNAs not only provide a new perspective for the understanding of disease mechanisms but also provide a new method for the diagnosis and treatment of disease [45]. More interestingly, we confirmed that the level of HOXA-AS2 was significantly increased in patients with PD and found that HOXA-AS2 expression was negatively associated with PGC-1α expression in a relatively large cohort. Additionally, the combination of HOXA-AS2 and PGC-1α in PBMCs showed higher accuracy in discriminating PD patients from healthy controls.

## Conclusion

In summary, this study provides convincing evidence that the upregulated lncRNA HOXA-AS2 promotes neuroinflammation by regulating microglial polarization in vitro and in vivo. Mechanistically, HOXA-AS2 acted as a modular scaffold of PRC2 and epigenetically silenced PGC-1α by regulating the methylation of PGC-1α promoters. Clinically, HOXA-AS2/PGC-1α might be a promising diagnostic indicator for PD. Our study indicates great value for HOXA-AS2 as a potential therapeutic target of neuroinflammation. Moreover, uncovering the role of the regulatory axis will not only increase our knowledge of lncRNA-regulated microglial polarization in PD and their underlying mechanisms but also help to develop more efficient strategies to treat PD.

## Supplementary Information

**Additional file 1: Table S1.** siRNAs sequence. **Table S2.** The list of primers. **Figure S1**. Relative expression of HOXA-AS2 in BV2 cells transfected with HOXA-AS2 overexpression plasmid or siRNAs. **Figure S2.** RNA-Protein Interaction Prediction database was used to analyze the binding potential of EZH2, SUZ12 and RbAp48 to HOXA-AS2.

## Data Availability

The key data are included in this published article and its supplementary information files. All microarray data have been deposited in the Gene Expression Omnibus (GEO GSE176167).

## Abbreviations

LncRNA: Long non-coding RNA
PRC: Polycomb repressive complex
PGC-1α: Peroxisome proliferator-activated receptor gamma coactivator-1α
PD: Parkinson’s disease
PBMCs: Peripheral blood mononuclear cells
DA: Dopaminergic
SN: Substantia nigra
RNA-seq: RNA-sequencing
RIP: RNA immunoprecipitation
ChIP: Chromatin immunoprecipitation
RT-qPCR: Real-time quantitative PCR
FISH: Fluorescence in situ hybridization
ICF: Immunocytofluorescence staining
ROC: Receiver operating characteristic
AUC: Area under the curve
LPS: Lipopolysaccharide
IL-4: Interleukin-4
IL-6: Interleukin-6
TNF-α: Tumor necrosis factor-α
Arg: Arginase
EZH2: Enhancer of zeste homolog 2
SUZ12: Suppressor of zest 12
RbAp48: Retinoblastoma-associated protein48
H3K27: Histone H3 at lysine 27
LSD1: Lysine-specific demethylase 1
NF-κB: Nuclear factor kappa B
