# Supplementary material for: LncRNA HOXA-AS2 regulates microglial polarization via recruitment of PRC2 and epigenetic modification of PGC-1α expression
Source: J Neuroinflammation. 2021 Sep 12;18:197. doi: 10.1186/s12974-021-02267-z (PMC8436538; doi:10.1186/s12974-021-02267-z)
Supplement: Supplementary file 1 — Additional file 1: Table S1. siRNAs sequence. Table S2. The list of primers. Figure S1. Relative expression of HOXA-AS2 in BV2 cells transfected with HOXA-AS2 overexpression plasmid or siRNAs. Figure S2. RNA-Protein Interaction Prediction database was used to analyze the binding potential of EZH2, SUZ12 and RbAp48 to HOXA-AS2. [file 12974_2021_2267_MOESM1_ESM.docx]

**Table S1: siRNAs sequence.**

| **siRNAs** | **sense sequence** | **anti-sense sequence** |
| --- | --- | --- |
| HOXA-AS2i-1 | 5'-GAGUUCAGCUCAAGUUGAACAUACA-3' | 5'-UGUAUGUUCAACUUGAGCUGAACUC-3' |
| HOXA-AS2i-2 | 5'-CAAGCUUGACAAGUUCAGCUCAA-3' | 5'-UUGAGCUGAACUCUUGUCAAGCUUG-3' |
| PGC-1αi | 5'-CCGCAAUUCUCCCUUGUAUTT-3' | 5'-AUACAAGGGAGAAUUGCGGTT-3' |
| Scr | 5'-UUCUCCGAACGUGUCACGUTT-3' | 5'-ACGUGACACGUUCGGAGAATT-3' |

**Table S2: The list of primers.**

| **RT-qPCR primers** | |  |
| --- | --- | --- |
|  | **Forward Primer** | **Reverse Primer** |
| HOXA-AS2-m | CCCGTAGGAAGAACCGATGA | TTTAGGCCTTCGCAGACAGC |
| HOXA-AS2-h | CCCGTAGGAAGAACCGATGA | TTTAGGCCTTCGCAGACAGC |
| PGC-1α-m | TATGGAGTGACATAGAGTGTGCT | CCACTTCAATCCACCCAGAAAG |
| PGC-1α-h | TAAACGACTCCGAGAACA | GACCCAAACATCATACCC |
| CD16 | TTTGGACACCCAGATGTTTCAG | GTCTTCCTTGAGCACCTGGATC |
| IL-6 | TAGTCCTCCTACCCCAATT | TTGGTCCTTAGCCACTCCTT |
| TNF-α | GCCTCCCTCTCATCAGTTCT | ACTTGGTGGTTTGCTACGAC |
| Arg | GAACACGGCAGTGGCTTTAAC | TGCTTAGCTCTGTCTGCTTTGC |
| Ym1 | TACTCCTCAGAACCGTCAGAT | CATTTCCTTCACCAGAACAC |
| CD206 | AAGATCTCATGGGCAACATCG | CTTGCCAGGATAGTAAATGAGCAAT |
| GAPDH-m | TGGTGAAGCAGGCATCTGAG | TGAAGTCGCAGGAGACAACC |
| GAPDH-h | CTCTGACTTCAACAGCGACACC | CTGTTGCTGTAGCCAAATTCGTT |
| **primers for ChIP** | |  |
|  | **Forward Primer** | **Reverse Primer** |
| HOXA-AS2 | AAGTTCAGGTGGCGCTTTGTCTA | ACGATTAAATACCATGCCGCAGA |
| PGC-1α | TCTGGTAAGGGTGTGCTGTG | AAAACTCCTCTGTGGCATGTG |


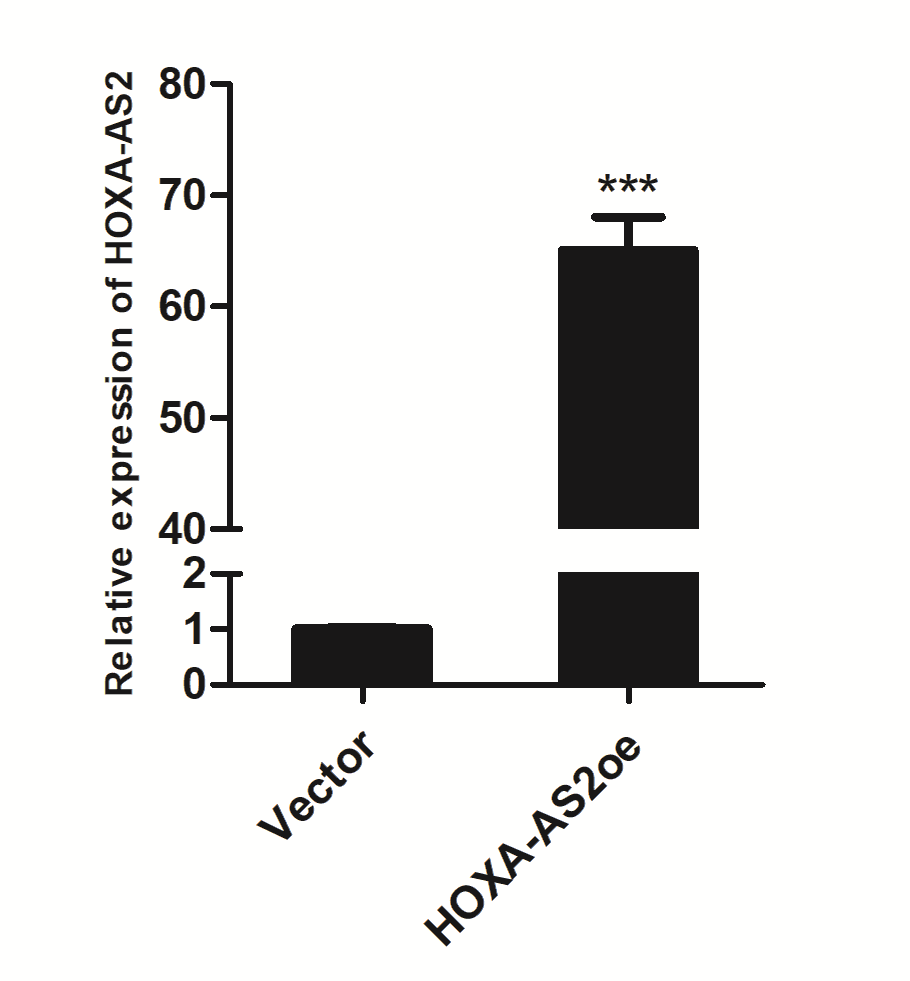

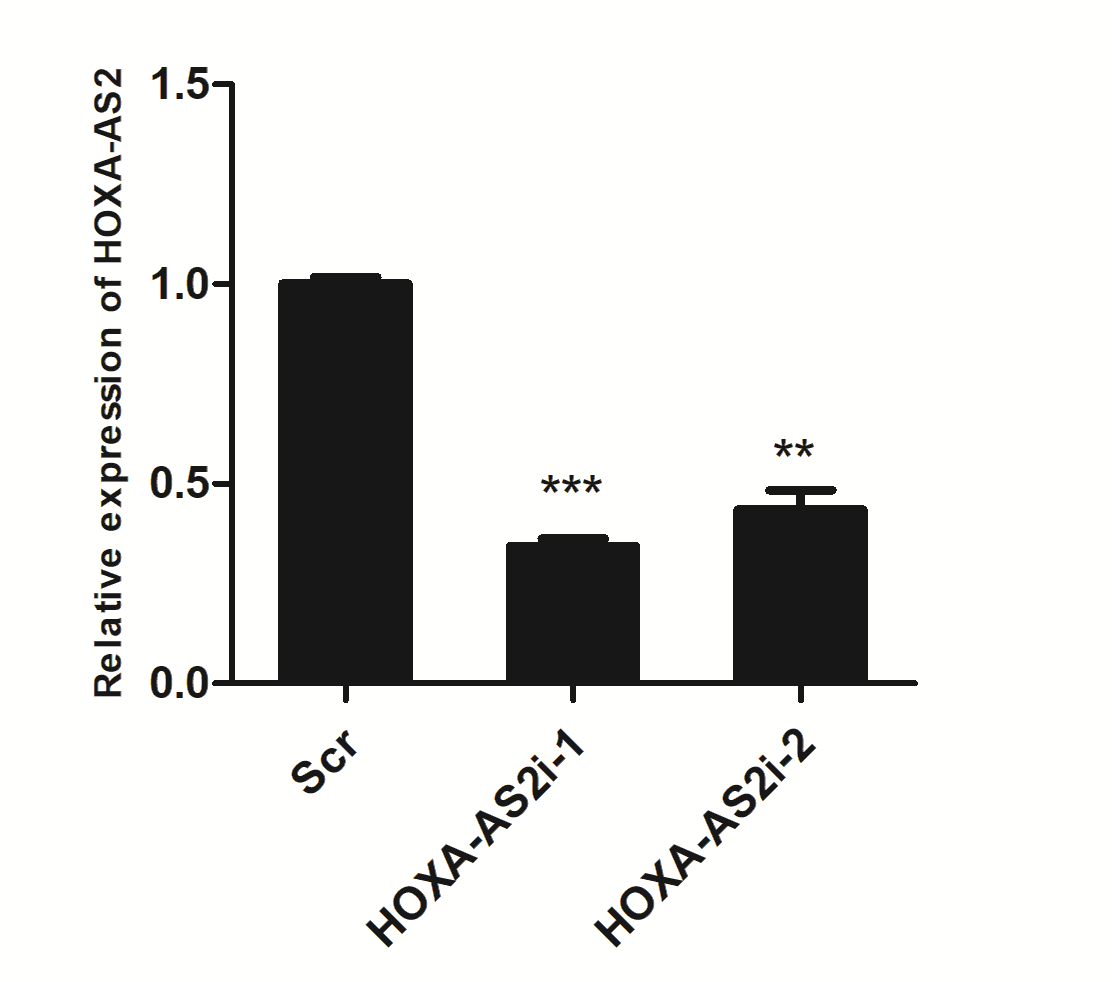


**Figure S1:** Relative expression of HOXA-AS2 in BV2 cells transfected with HOXA-AS2 overexpression plasmid or siRNAs.


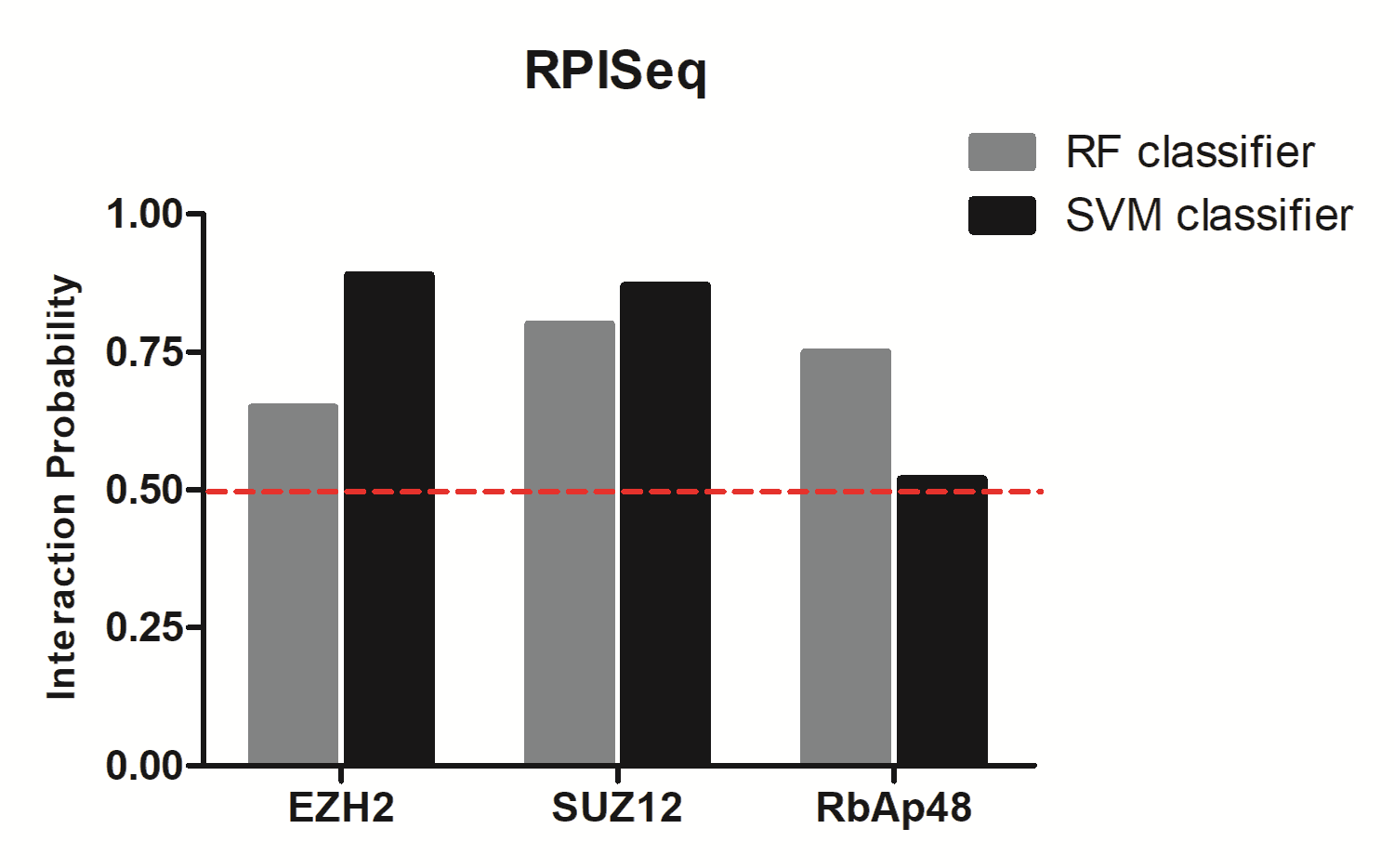


**Figure S2:** RNA-Protein Interaction Prediction database was used to analyze the binding potential of EZH2, SUZ12 and RbAp48 to HOXA-AS2.
